# Integration of Diagnostic Lung Ultrasound Into Clinical Practice by Hospitalists in an Academic Medical Center: A Retrospective Chart Review

**DOI:** 10.7759/cureus.69796

**Published:** 2024-09-20

**Authors:** John-David Slaugh, Meltiady Issa, Eric Grimm, Antonio J Calderon, Solomon Sindelar, Reed Van Hook, Lauren McBeth, Anna Maw

**Affiliations:** 1 Internal Medicine, University of Colorado Anschutz Medical Campus, Aurora, USA; 2 Hospital Internal Medicine, Mayo Clinic, Rochester, USA; 3 Hospital Medicine, University of Colorado Anschutz Medical Campus, Aurora, USA; 4 Medicine, University of Colorado Anschutz Medical Campus, Aurora, USA

**Keywords:** decision-making, covid-19, point of care, hospitalists, lung ultrasound

## Abstract

Background

Point-of-care lung ultrasound (LUS) is a guideline-recommended imaging modality that has been shown to be more accurate than chest radiography for multiple causes of dyspnea. This study was conducted to understand the impact of LUS on real-world clinical decision-making among hospitalists.

Methods

A retrospective chart review was conducted of patients who received a LUS while hospitalized at a quaternary care academic medical center between July 2020 and June 2022. Data was extracted from the electronic health record (EHR) into a standardized REDCap form. Cases were defined as patients who had received a LUS that (1) had images archived and accessible to viewing through the EHR and (2) had an imaging report documented in the EHR.

Results

Of the 820 LUSs reviewed, 297 (36.2%) were performed to evaluate for appropriateness of thoracentesis, 205 (25%) for diagnosing or monitoring of pneumonia related to COVID-19, 169 (20.6%) for volume status assessment, 136 (16.6%) for worsening respiratory status, 114 (13.9%) for monitoring pleural effusions, 64 (7.8%) for diagnosing or monitoring of pneumonia not related to COVID-19, and 12 (1.5%) for monitoring of diuresis. Documentation was sufficient to determine clinical decision-making in 730 (89%) of LUSs reviewed, 739 (90.1%) were considered to be diagnostically useful, and 327 (39.9%) changed management.

Conclusions

These findings suggest LUS was diagnostically useful and routinely changed management in hospitalist practice. Further, documentation in the EHR was sufficient to allow for the evaluation of real-world clinical decision-making using LUS, which is an important gap in both the education and health services research literature.

## Introduction

Point-of-care lung ultrasound (LUS), an ultrasound of the lung that is performed at the bedside by a clinician, is an imaging modality that can be more accurate than physical exam maneuvers or chest radiography for multiple common causes of dyspnea including pneumonia, pulmonary edema, pleural effusion, and pneumothorax [1-7]. LUS is an American College of Physicians' guideline-recommended test that increases the accuracy of diagnosis in acutely dyspneic patients relative to standard diagnostic approaches while also avoiding exposing patients to ionizing radiation [1]. Given the growing evidence that it expedites accurate diagnosis, multiple professional societies now endorse LUS use in acute care settings [1,8-10].

Despite its many advantages, the adoption of LUS among internal medicine hospitalists remains low [11]. Previously, low adoption has been attributed to a lack of access to equipment and training [12]. However, with the increasing affordability and portability of ultrasound devices as well as increased opportunities for training, the barriers for adoption are likely shifting.

In 2020, we performed a pilot study aimed at understanding the implementation and adoption of LUS by hospitalists [13] within the setting of the COVID-19 pandemic. We studied the adoption of LUS through the lens of diffusion of innovation (DOI) theory which aims to explain how new ideas, technologies, or practices spread through a social system over time [14,15]. We used DOI theory to help identify barriers to implementation and develop strategies to facilitate use. Performed two years after the initiation of LUS implementation, the objective of this retrospective chart review was to better understand current clinician adoption, diagnostic utility, and the effect on patient management of LUS in real-world hospitalist practice.

This article was previously presented as a meeting poster at the Society of General Internal Medicine Mountain West Regional Meeting on November 4, 2022.

## Materials and methods

This retrospective chart review was determined to be exempt by the University of Colorado Institutional Review Board because it was a secondary analysis of de-identified patient data. Inclusion criteria for this study were all patient encounters in which a hospitalist-specific LUS order was placed between the dates of June 2020 and March 2022. All LUS images were acquired using handheld devices exclusively. Data extracted from the EHR was subsequently imported into a REDCap database [16]. Cases were defined as patients who had received LUS that (1) had images archived and accessible to viewing through the EHR and (2) had a LUS imaging report documented in the EHR. LUS exams not meeting this criteria were excluded from this study.

The principal investigator (PI) trained four members of the research team in data extraction from the EHR. She supervised the first five chart reviews in real time and then checked the first five chart reviews conducted independently, giving feedback to each team member to ensure consistency in data extraction. Data from clinical documentation describing the indication for LUS, the LUS imaging report, and the role LUS played in clinical decision-making were manually extracted by these four members of the research team. When the data extractor deemed there was insufficient documentation to determine the role LUS played in clinical decision-making, this was also documented. The PI randomly selected 10% of encounters and spot-checked the REDCap data forms to ensure they were being filled out with consistency and as intended.

Data extractors adjudicated LUS diagnostic and decision-making outcomes of interest by reviewing the clinical documentation and using a pre-specified criteria (Table 1). LUS exams were considered diagnostically useful if they were documented in the EHR as follows: (1) changing the relative pretest probability of a diagnosis, (2) offering sufficient information for the monitoring of a disease process, or (3) offering sufficient information to decide whether thoracentesis was indicated. LUS was marked as confirming a diagnosis if LUS findings (1) were consistent with a previously documented diagnostic impression or (2) confirmed the presence of a pleural effusion (whether amenable or non-amenable) for thoracentesis. LUS was marked as changing the diagnostic impression if (1) the LUS was not consistent with the original diagnostic impression, (2) the LUS revealed a new diagnosis not considered prior to the exam, or (3) evaluating for thoracentesis revealed a fluid pocket not amenable for thoracentesis or no fluid pocket at all. LUS was marked as changing management if (1) a therapy was given or withheld from a patient based on LUS findings, (2) another imaging modality was ordered based on LUS findings, (3) another specialty or consulting service was asked to evaluate the patient based on LUS findings, or (4) a thoracentesis was performed based on LUS findings. Descriptive statistics were used to describe the results.

**Table 1:** LUS diagnostic and decision-making outcomes criteria LUS: lung ultrasound

	Diagnostically useful	Confirms a diagnosis	Changes the diagnostic impression	Changes management
Criteria	Changes the relative pretest probability of a diagnosis	Consistent with a previously documented diagnostic impression	Not consistent with the original diagnostic impression	A therapy is given or withheld based on LUS findings
	Offers sufficient information for the monitoring of a disease process	Confirms the presence of a pleural effusion (whether amenable or non-amenable) for thoracentesis	Reveals a new diagnosis not considered prior to the exam	Another imaging modality is ordered based on LUS findings
	Offers sufficient information to decide whether thoracentesis was indicated		Thoracentesis evaluation reveals a fluid pocket not amenable for thoracentesis or no fluid pocket at all	Another specialty or consulting service is asked to evaluate the patient based on LUS findings
				A thoracentesis was performed based on LUS findings

## Results

Cohort demographics were comprised of 46.3% (275/594) males, 56.4% (335/594) White, and 75.3% (447/594) non-Hispanic (Table 2) which reflects the patient population at our specific institution. Most clinicians ordering LUSs were physicians at 56.3% (45/80) followed by physician assistants at 31.3% (25/80) with the remainder being nurse practitioners at 11.3% (9/80). Clinicians who performed or supervised image acquisition and interpreted the LUS images were predominantly physicians at 85.7% (12/14), followed by physician assistants at 14.3% (2/14) (Table 3).

**Table 2:** Patient demographics The data has been represented as N and %

	Number of patients, N=594	Percent of patients
Patient sex		
Male	275	46.3%
Female	319	54.7%
Patient race		
American Indian or Alaska Native	8	1.3%
Black or African American	83	14%
Native Hawaiian	2	0.3%
Native Hawaiian and Other Pacific Islander	1	0.2%
Other Asian	32	5.4%
Other Pacific Islander	2	0.3%
Vietnamese	1	0.2%
White or Caucasian	335	56.4%
Other	124	20.9%
More than one race	2	0.3%
Patient refused	4	0.7%
Patient ethnicity		
Hispanic	143	24.1%
Non-Hispanic	447	75.3%
Patient unable to answer	3	0.5%
Unknown	1	0.2%

**Table 3:** LUS utilization by clinician training and sex The data has been represented as N and % LUS: lung ultrasound

	Authorizing order clinicians, N=80	Finalizing order clinicians, N=14
Clinician type		
Physician	45 (56.3%)	12 (85.7%)
Physician assistant	25 (31.3%)	2 (14.3%)
Nurse practitioner	9 (11.3%)	0 (0%)
Fellow	1 (1.3%)	0 (0%)
Clinician sex		
Male	33 (41.3%)	9 (64.3%)
Female	47 (58.8%)	5 (35.7%)

**Figure 1:**
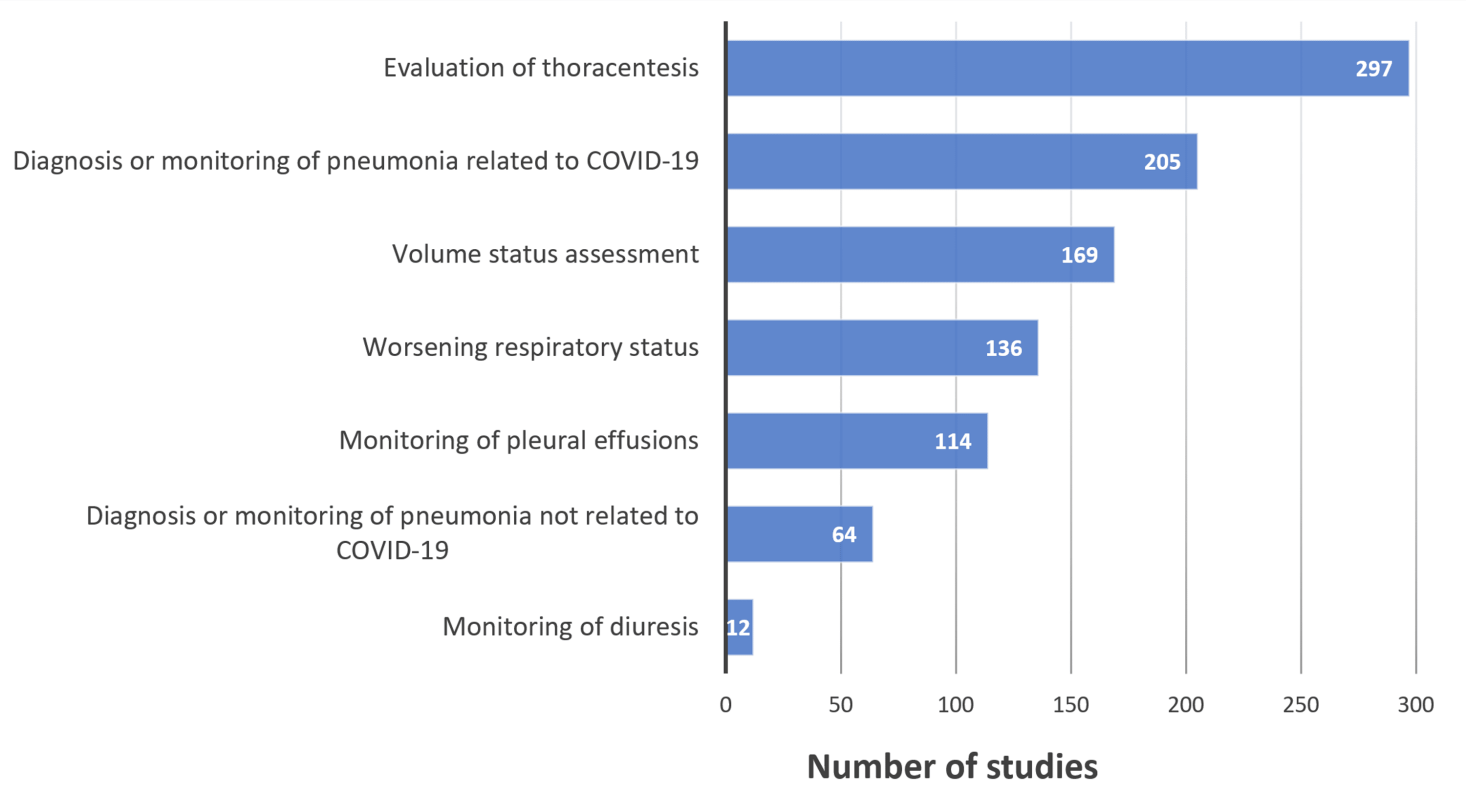
Indications for LUS Data has been presented as N LUS: lung ultrasound

Of the 820 LUSs reviewed, 297 were performed to evaluate for appropriateness of thoracentesis, 205 for diagnosing or monitoring pneumonia related to COVID-19, 169 for volume status assessment (evaluating for the presence of sonographic b-lines or pleural effusions), 136 for worsening respiratory status, 114 for monitoring pleural effusions, 64 for diagnosing or monitoring of pneumonia not related to COVID-19, and 12 for monitoring of diuresis (evaluating for a decrease in the number of sonographic b-lines or size of pleural effusions) (Figure 1). Around 79% (648/820) of LUSs were ordered for a single indication, 20.4% (167/820) were ordered for two indications, and the remaining 0.6% (5/820) were ordered for three indications (Table 4).

**Table 4:** Number of indications selected per chart Data has been represented as N and % LUS: lung ultrasound

Number of indications selected per LUS	Number of charts, N=820
1 indication	648 (79%)
2 indications	167 (20.4%)
3 indications	5 (0.6%)

**Figure 2:**
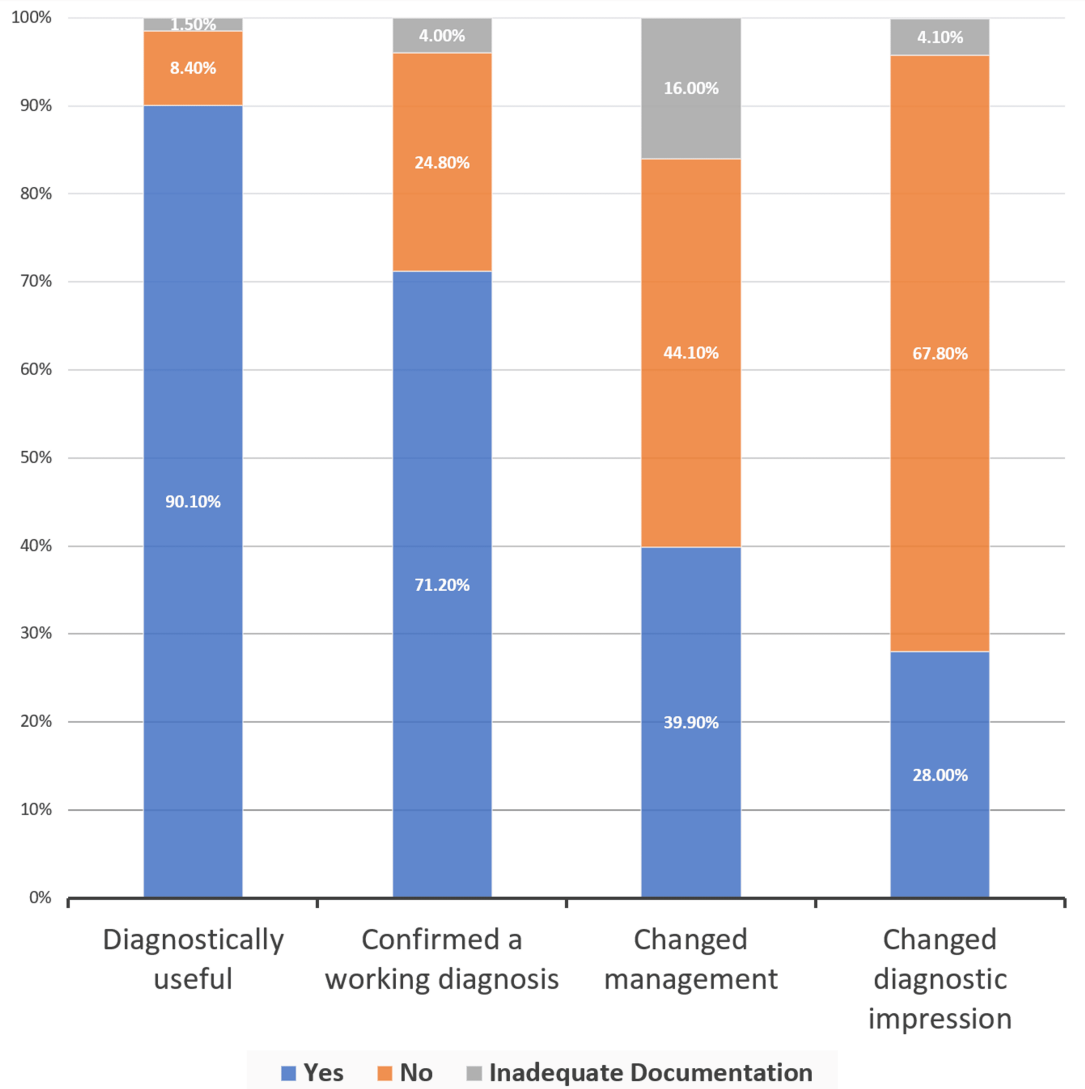
Parameters evaluating LUS outcomes* *Rounding may cause percentage totals to not equal exactly 100% Data has been presented as % LUS: lung ultrasound

With regard to utility in clinical decision-making, 90.1% (739/820) were determined to be diagnostically useful (i.e., LUS results changed relative pretest probability), and 39.9% (327/820) changed management (i.e., thoracentesis was performed, or diuretics were given based on LUS findings). LUS confirmed a working diagnosis in 71.2% (584/820) of cases and changed diagnostic impression in 28% (230/820) of cases (Figure 2). These data were broken down further to study the impact on clinical decision-making for each LUS indication (Table 5). For example, LUS changed management in 61.6% (183/297) of cases when evaluating for thoracentesis but only in 14.6% (30/205) of cases of pneumonia related to COVID-19. Notably, LUS was considered diagnostically useful, confirmed a working diagnosis, and changed diagnostic impression for each LUS for which monitoring for diuresis was the indication (12/12).

**Table 5:** Impact on clinical decision-making Data has been presented at N and % LUS: lung ultrasound

LUS indication	Diagnostically useful		Confirmed a working diagnosis		Changed management		Changed diagnostic impression	
	Yes N (%)	Insufficient documentation N (%)	Yes N (%)	Insufficient documentation N (%)	Yes N (%)	Insufficient documentation N (%)	Yes N (%)	Insufficient documentation N (%)
Evaluation for thoracentesis, n=297	276 (92.9%)	2 (0.7%)	216 (72.7%)	17 (5.7%)	183 (61.6%)	3 (1%)	83 (27.9%)	17 (5.7%)
Pneumonia not related to COVID-19, n=64	62 (96.9%)	0 (0%)	39 (60.9%)	1 (1.6%)	20 (31.3%)	8 (12.5%)	29 (45.3%)	1 (1.6%)
Pneumonia related to COVID-19, n=205	182 (88.8%)	2 (1%)	165 (80.5%)	5 (2.4%)	30 (14.6%)	91 (44.4%)	39 (19%)	5 (2.4%)
Volume status assessment, n=169	160 (94.7%)	2 (1.2%)	108 (63.9%)	5 (2.7%)	76 (45%)	17 (10.1%)	57 (33.7%)	6 (3.6%)
Worsening respiratory status, n=136	117 (86%)	5 (3.7%)	75 (55.1%)	6 (4.4%)	46 (33.8%)	25 (18.4%)	57 (41.9%)	6 (4.4%)
Monitoring of pleural effusions, n=114	83 (72.8%)	1 (0.9%)	86 (75.4%)	0 (0%)	42 (36.8%)	5 (4.4%)	35 (30.7%)	0 (0%)
Monitoring of diuresis	12 (100%)	0 (0%)	12 (100%)	0 (0%)	5 (41.7%)	0 (0%)	12 (100%)	0 (0%)

Data extractors deemed documentation adequate to evaluate clinical decision-making using LUS in 89% (730/820) of exams reviewed. Insufficient documentation was most common when attempting to determine whether LUS changed management in pneumonia related to COVID-19, comprising 61% (91/150) of insufficient data occurrences.

## Discussion

These data demonstrate that LUS was considered diagnostically useful and changed management in 90.1% and 39.9% of archived LUSs, respectively. These results are consistent with other studies performed with more restrictive inclusion criteria [17] and suggest that overall, LUS is a useful clinical tool for hospitalists in real-world practice. Another notable study finding is that the documentation of clinical decision-making using LUS images was present in 89% of cases in which LUS images were uploaded to the EHR. This suggests the current archiving and documentation workflow at our hospital may allow for the evaluation of multiple patient and health system outcomes using LUS in real-world practice including downstream testing and intensive care unit transfers. Evaluation of these outcomes is cited as an evidence gap in current professional society guidelines [1].

To our knowledge, the demonstration of the utility of such a developed archive and documentation infrastructure to understand real-world decision-making using LUS by hospitalists is a unique contribution to the literature. In future work, we plan to leverage EHR data to evaluate real-world LUS application using more rapid and lower-burden methods including data mining of clinical documentation to further automate data extraction. Automated data extraction will facilitate a more timely evaluation of LUS use and allow for interval quality improvement efforts based on up-to-date information.

These data suggest that even in cases in which management was unchanged by LUS, diagnostic uncertainty was often reduced. Future studies should explore potential benefits to reducing diagnostic uncertainty in cases in which management is not changed. These potential benefits include expediting care, reducing clinician cognitive load, improving clinician experience, and reducing additional, less patient-centric tests like chest X-rays and computed tomography [18,19].

Another notable finding is that 61% (91/150) of LUS exams that lacked sufficient documentation in one of the four categories and 69% (91/131) of exams that lacked sufficient documentation to determine whether exam results changed clinical management were in the exams performed to assess patients with COVID-19 pneumonia. This may suggest that clinicians were not using LUS in their clinical decision-making, which is consistent with the qualitative themes found in our previous study [13].

An additional notable finding is that LUS was infrequently used to monitor diuresis. This is an important observation because there is substantial evidence that monitoring b-lines in patients with heart failure is a more accurate measure of volume status changes than traditional methods (i.e., daily weights, ins, and outs) and that the number of b-lines on discharge correlates with death and rehospitalization [20]. There is also randomized controlled trial data demonstrating that LUS-guided diuresis decreases the length of stay in patients with heart failure [21,22]. Given that heart failure is the most common admitting diagnosis in older adults [23], increasing the adoption of LUS for this indication is a high-yield implementation target. This insight provided by our evaluation demonstrates the value of interval analyses to understand how to prioritize implementation efforts.

One of the strengths of this study is the large number of cases reviewed, facilitating a more complete view of the real-world use of LUS within an academic hospitalist group. Additionally, to our knowledge, this is the first study to report clinical decision-making in real-world practice, using handheld devices by hospitalists. This is an important gap in the literature given the growing use of handheld ultrasound devices by clinicians [24]. Limitations of this study include that it was performed at a single hospital, which limits the generalizability of our findings. Additionally, we were unable to capture the number or characteristics of phantom scans: scans performed and used for clinical decision-making but not ordered through the EHR and therefore not traceable. Finally, because data were not collected prospectively, members of the research team had to interpret clinician documentation to categorize EHR data which may have resulted in some amount of error. 

## Conclusions

These results suggest that the use of LUS by hospitalists is diagnostically useful and often changes management. It also demonstrates the utility of integrating point-of-care ultrasound images and documentation into the EHR within hospital medicine groups. Further studies should explore the potential advantages of LUS in reducing diagnostic uncertainty and its impact on clinicians' cognitive load and job satisfaction.
